# Carbon and Nitrogen Speciation in N-poor C-O-H-N Fluids at 6.3 GPa and 1100–1400 °C

**DOI:** 10.1038/s41598-017-00679-7

**Published:** 2017-04-06

**Authors:** Alexander G. Sokol, Anatoly A. Tomilenko, Taras A. Bul’bak, Galina A. Palyanova, Ivan A. Sokol, Yury N. Palyanov

**Affiliations:** 1 V.S. Sobolev Institute of Geology and Mineralogy, Siberian Branch of the Russian Academy of Sciences, ave. Koptyuga 3, Novosibirsk, 630090 Russia; 2 Novosibirsk State University, str. Pirogova 2, Novosibirsk, 630090 Russia

## Abstract

Deep carbon and nitrogen cycles played a critical role in the evolution of the Earth. Here we report on successful studying of speciation in C-O-H-N systems with low nitrogen contents at 6.3 GPa and 1100 to 1400 °C. At *f*O_2_ near Fe–FeO (IW) equilibrium, the synthesised fluids contain more than thirty species. Among them, CH_4_, C_2_H_6_, C_3_H_8_ and C_4_H_10_ are main carbon species. All carbon species, except for C_1_-C_4_ alkanes and alcohols, occur in negligible amounts in the fluids generated in systems with low H_2_O, but С_15_-С_18_ alkanes are slightly higher and oxygenated hydrocarbons are more diverse at higher temperatures and H_2_O concentrations. At a higher oxygen fugacity of +2.5 Δlog *f*O_2_ (IW), the fluids almost lack methane and contain about 1 rel.% C_2_-C_4_ alkanes, as well as fractions of percent of C_15–18_ alkanes and notable contents of alcohols and carboxylic acids. Methanimine (CH_3_N) is inferred to be the main nitrogen species in N-poor reduced fluids. Therefore, the behaviour of CH_3_N may control the nitrogen cycle in N-poor peridotitic mantle. Oxidation of fluids strongly reduces the concentration of CH_4_ and bulk carbon. However, higher alkanes, alcohols, and carboxylic acids can resist oxidation and should remain stable in mantle hydrous magmas.

## Introduction

Abiotic hydrocarbons (HCs) and ammonia have been important agents in the Earth’s carbon and nitrogen cycles. Their stability in different tectonic settings in the course of geodynamic evolution could control the habitability of the planet, the amounts of carbon and nitrogen migrating in fluids, the processes of redox melting and freezing, and the formation of diamonds^1–6^. There is a wealth of evidence for the existence of hydrocarbons in the mantle. Namely, Sugisaki and Mimura (ref. 7) studied a collection of 227 samples from fifty localities throughout the world and revealed heavy hydrocarbons (n-alkanes) in mantle-derived rocks, such as peridotites in ophiolite sequences or peridotite xenoliths in alkali basalts, but did not find any in gabbro and granite. HCs in inclusions hosted by diamond and other minerals in mantle xenoliths were reported from the Siberian craton^8–11^. As a recent find, we can cite a thin fluid jacket of CH_4_ around inclusions of solidified iron-nickel-carbon-sulfur melt in large, exceptional gem (like the Cullinan, Constellation, and Koh-i-Noor) sublithospheric diamonds reported by Smith *et al*. (ref. 12).

The genesis of hydrocarbons in the mantle remains unclear. They might be a mixture of HCs which were recycled or delivered by meteorites and comets to the early (primeval) Earth, or synthesised by the Fischer-Tropsch reaction^7^. The contribution of two former sources may be, however, limited: HCs may oxidise during subduction and can hardly survive the high impact temperatures. The composition of hydrocarbons may vary. For instance, HCs heavier than methane were inferred^13–17^ to be energetically favoured at the deep mantle *P-T* conditions. As for nitrogen in reduced mantle, there is a consensus that most of it resides in ammonia and NH_4_^+^ which substitutes for alkali cations in phengite, Cr-bearing clinopyroxene and other silicates^2, 18, 19^.

The behaviour of carbon and nitrogen species in mantle fluids depends on oxygen fugacity (*f*O_2_). Estimates for continental lithospheric mantle (CLM) within the Kaapvaal^20^, Slave^21^ and Siberian^22, 23^ cratons show that *f*O_2_ generally decreases depthward from +1 Δlog *f*O_2_ FMQ (Fayalite-Magnetite-Quartz oxygen buffer) at 100 km (~3.2 GPa) to -4 Δlog *f*O_2_ FMQ at a depth of 220 km (~7 GPa). The *f*O_2_ values at the CLM base about 200 km below the surface may vary from IW + 1 to IW + 3 log units^1^ and the temperatures at this depth may range within 1100–1400 °С^20–23^. According to thermodynamic calculations, lower alkanes (especially methane and ethane) and ammonia are expected to be stable under *f*O_2_ conditions of the CLM^3, 24–26^, but they almost disappear from fluids at *f*O_2_ reaching CW (the maximum H_2_O content in C-O-H fluids). Moreover, the silicate environment can influence the pH of deep H_2_O-bearing fluids and thus the carbon and nitrogen speciation^6, 26, 27^.

Recent decades have brought much progress in experimental techniques with *f*O_2_ and *f*H_2_ buffering used to predict potential compositions of hydrocarbon fluids at mantle pressures and temperatures. Carbon-saturated C-O-H fluids in reduced systems were studied by quenching experiments and chromatography at 2.0–3.5 GPa and 1000–1300 °С^28, 29^, as well as at 5.7–6.3 GPa and 1200–1600 °С^30, 31^. Carbon concentrations in strongly reduced fluids synthesised under these *Р-Т*-*f*O_2_ conditions are commonly related as CH_4_ > C_2_H_6_ > C_3_H_8_ (other HCs being ≪1%) and agree well with calculations^20, 25, 28, 32^. The concentrations of lower alkanes at pressures from 4 to 7 GPa and typical CLM temperatures^25, 32, 33^ vary only little, but the precise contents of other hydrocarbons remain unknown. Among nitrogen species, ammonia was reported^3^ to predominate in nitrogen rich N-O-H fluids entrapped in quartz and olivine at 0.2–3.5 GPa, 600–1400 °C and *f*O_2_ at the Fe-FeO equilibrium, as well as in ultra-reduced nitrogen-rich C-O-H-N fluids at 5.5–7.8 GPa and 1100–1500 °C, as shown by our recent experiments^34^. In upper mantle aqueous fluids under reducing conditions, nitrogen is expected to occur mainly as ammonium (NH_4_^+^)^26^.

In this study we apply quenching experiments with buffered hydrogen fugacity to study (i) the stability of different carbon and nitrogen species in N-poor C-O-H-N fluids at 6.3 GPa, 1100–1400 °C and *f*O_2_ about or slightly below IW corresponding to the conditions at the СLM base (~200 km depth) near the boundary with the asthenosphere, and (ii) the relative stability of HCs and N-bearing compounds at *f*O_2_ IW +2.5 log units corresponding to reactions of reduced C-O-H-N fluids with oxidised lithosphere. The estimates^25, 32, 33^ that the fluid composition does not depend much on pressure within 4–7 GPa allows us to extrapolate the results onto the upper mantle pressure range.

## Results

### Effect of cooling rate on behaviour of C-O-H-N fluids

Isobaric cooling can change the compositions of fluids as a result of back reactions. In this respect, it is critical for the fluids to cool down at rates sufficient for precluding back reactions and holding the equilibrium composition consistent with the target temperature and run duration. Changes in the species composition of fluids were studied previously in 2.4 GPa experiments in the C-O-H system synthesised at 1000 °C and cooling at rates from 0.3 to 120 deg/s at buffered *f*O_2_^29^. The potential effect was monitored against concentration changes of species involved in the C_2_H_6_ + H_2_ → 2CH_4_ reaction, and back reactions were shown to stop completely only at relatively rapid cooling of 120 deg/s. The fluids cooling at slower rates contained less hydrogen (2 to 4 mol.%) and, correspondingly, more CH_4_. The closure temperature for the C-O-H system equilibration was suggested to be <800 °C^29^. Earlier we^31^ investigated the effect of cooling rates in the range of 1 to 200 deg/s on the composition of C-O-H fluids synthesised at 6.3 GPa and 1600 °C in 15 hr-long runs. Comparison of fluids generated from the same starting materials but cooled at different rates showed CH_4_ and C_2_H_6_ to increase from 2–3 to 9–10 mole % and from 1 to 3 mole %, respectively, as cooling slowed down from 200 to 27 deg/s. Further deceleration to 1 deg/s did not change the concentration of CH_4_, but led to C_2_H_6_ increase from 3 to 4–5 mol.%. Therefore, synthesis of hydrocarbons most likely was due to back reactions of hydrogen with graphite during slow cooling, as we suggested^31^. Meanwhile, the changes of nitrogen speciation in O-H-N fluids at cooling rates from 70 to 140 deg/s in the forward and reversal experiments of Li and Keppler (ref. 3) were negligible, if any.

In this study we have performed three 2-hr long runs at 1400 °C, with quenching at 200 deg/s and slow cooling at 1 deg/s (Tables 1–3). The normalised peak areas for particular species in GC-MS spectra showed slight variations in lower alkanes in slowly cooling fluids with similar amounts of water (24–31 rel.%). The CH_4_/C_2_H_6_ ratio was above and below 1 at rapid and slow cooling, respectively. Cooling at 200 times slower rates did not cause notable changes to CH_4_, C_2_H_6_, C_3_H_8_ and C_4_H_10_ concentrations (Table 2) but led to about ten-fold increase of C_15_ to C_19_ alkanes (from ~0.01 to ~0.1) and formation of minor amounts (within ~0.5 rel.%) of olefins, arenes, and oxygenated hydrocarbons: aldehydes, ketones, and carboxylic acids (specifically, 0.2 rel.% acetic acid and higher concentrations of acids with C_10._ C_13_ and C_15_). Note especially that slow cooling resulted in the formation of more methanimine (CH_3_N), at the same *P-T*-τ conditions as during quenching (Table 2).

**Table 1:** Starting compositions (mg). Microscopic amounts of nitrogen in the capsules came from air.

Run^#^	Graphite	Docosane (C_22_H_46_)	Stearic acid (C_18_H_36_O_2_)
1761_2_3	18.7	1.8	—
1769_2_2	8	0.3	0.3
1315_3_5	26.2	—	3.1
1751_2_2	8.8	—	0.9
1751_2_3	9	0.6	—
1746_2_3	9.4	0.7	—
1746_2_2	8.4	—	0.9
1780_2_2	7.6	—	0.7
1780_2_3	8.1	0.8	—
1780_2_4	8.3	—	1.0
1016_7_2	8.2	—	0.5
1016_7_4	8.8	0.6	—
1753_2_3	18.6	—	1.7
1898_2_1	7.1	—	0.5
1898_2_3	7.8	0.3	0.3
888_7_1	23	1.9	—
889_7_1	23.7	1.4	—
1720_2_2	17.7	—	3.2
1727_2_2	17.8	3.9	—
1019_7_1	8.1	—	0.7
1019_7_3	7.3	0.4	0.3

**Table 2:** Experimental conditions and concentrations of main species (rel.%) in quenched C-O-H-N fluids synthesised at 6.3 GPa.

Run^#^	Starting composition*	Capsule	Buffer *f*H_2_	Time (h)	T (°C)	H_2_O	CH_4_	C_2_H_6_	C_3_H_8_	C_4_H_10_	CH_3_N/(CH_3_N + N_2_)	Calc. *f*O_2_
1761_2_3	Docosane	Pt	MMO	0.017	1100	11	10	74	5.2	0.2	1.00	—
1769_2_2	Stearic acid + docosane	Pt	MMO	7	1100	16	35	31	14	0.2	0.22	−10.8
1315_3_5	Stearic acid	Pt	—	7	1200	93	2.9	0.7	0.4	1.0	0.03	−8.5
1751_2_2	Stearic acid	Au	MMO	7	1200	41	37	18	2.7	0.2	0.99	−10.8
1751_2_3	Docosane	Au	MMO	7	1200	9.6	50	31	8.1	0.4	0.99	−15.4
1746_2_3	Docosane	Pt	MMO	2	1300	11	26	38	21	1.7	0.80	−9.0
1746_2_2	Stearic acid	Pt	MMO	2	1300	39	23	28	5.0	1.8	0.98	−10.5
1780_2_2	Docosane	Pt	MMO	7	1300	4.2	27	36	24	5.7	0.95	−9.3
1780_2_3	Stearic acid	Pt	MMO	7	1300	55	13	24	4.9	0.9	Only N_2_	−8.5
1780_2_4	Stearic acid***	Pt	MMO	7	1300	6.9	5.2	4.2	0.2	0.02	Only N_2_	—
1016_7_2	Stearic acid	Pt	MMO	0.017	1400	25	11	31	23	3.6	0.93	—
1016_7_4	Docosane	Pt	MMO	0.017	1400	32	11	31	17	4.2	0.93****	—
1753_2_3	Stearic acid	Pt	MMO	2	1400	31	38	20	8.6	2.2	Only CH_3_N	−10.6
1898_2_1	Stearic acid	Pt	MMO	2	1400**	30	16	32	15	2.2	1.00	−10.5
1898_2_3	Stearic acid + docosane	Pt	MMO	2	1400**	24	17	35	14	2.2	1.00	−10.9
888_7_1	Docosane	Pt	MMO	2	1400**	4.4	23	71	0.8	1.0	1.00	—
889_7_1	Docosane	Pt	MMO	7	1400	0.7	51	38	8.7	0.6	0.69	—
1720_2_2	Stearic acid	Pt	—	7	1400	96	0.06	1.4	0.08	0.8	Only N_2_	−6.0
1727_2_2	Docosane	Pt	MMO	7	1400	11	22	34	4.9	3.5	0.72	−8.8
1019_7_1	Stearic acid	Pt	MMO	10	1400	52	18	25	2.7	1.5	0.98	−8.1
1019_7_3	Stearic acid + docosane	Pt	MMO	10	1400	26	18	35	14	3.3	0.99	−11.0

Full compositions are given in Supplementary Table 1. MMO is Mo-MoO_2_ buffer. CH_3_N/(CH_3_N + N_2_) is normalised peak area ratio A(17 + 29 m/z)/(A(17 + 29 m/z) + A(28 m/z)). *All starting compositions include graphite, at 10/1 to fluid generating material. **Fluid cooling at 1 deg/s. ***Very little gas released upon capsule opening. ****NH_3_ is main nitrogen species.

**Table 3:** Representative analyses of quenched and slow cooling C-O-H-N fluids (rel.%) obtained in 2-hr runs at 6.3 GPa and 1400 °C.

Run^#^		1753_2_3	1898_2_3	1898_2_1
Capsule		Pt	Pt	Pt
Starting composition^*^		Stearic acid	Stearic acid + docosane	Stearic acid
Cooling rate		200 deg/s	1 deg/s	1 deg/s
Water		30.8	24.3	29.7
Alkanes	CH_4_	37.6	17.0	16.1
	C_2_H_6_	20.0	34.5	31.9
	C_3_H_8_	8.6	14.3	14.9
	C_4_H_10_	2.2	2.1	2.2
	C_5_H_12_	0.08	0.1	0.09
	C_6_-C_15_	≤0.002	≤0.04	≤0.03
	C_15_-C_19_^**^	≤0.01	≤0.3	≤0.1
Olefins		—	≤0.06	≤0.02
Arenes		—	≤0.008	≤0.02
Alcohols and ethers		≤0.1	≤0.1	≤0.08
Aldehydes		—	≤0.07	≤0.08
Ketones		≤0.005	≤0.03	≤0.05
Carboxylic acid		—	≤0.5	≤0.05
Furans		≤0.001	≤0.01	≤0.03
Nitrogen species				
N_2_		—	0.05	0.01
CH_3_N		0.5	>1	>1

*All samples contained graphite. **The quoted concentrations are maximum for a species of the respective group. All GC-MS data are given in Supplementary Table 1.

Therefore, back reactions in slowly cooling C-O-H-N fluids have different directions at the pressures 2.4 GPa and 6.3 GPa. Cooling leads mainly to synthesis of CH_4_ and consumption of alkanes by the reaction C_2_H_6_ + H_2_ = 2CH_4_ at the lower pressure^29^, but CH_4_ either increases^31^ or decreases (this study) slightly at 6.3 GPa, while the ratios of CH_4_ to C_2_H_6_, C_3_H_8_ and C_4_H_10_ at high and low cooling rates scatter within a reasonable error. However, slow cooling fluids obtained in this study are remarkable by higher concentrations of species that trace the back reactions (some olefins, arenes, aldehydes, ketones, and carboxylic acids). Thus, our and published data, including our previous results for 6.3 GPa and 1600 °C^31^, indicate that only cooling from 1100–1400 °C to room temperature at a rate of 200 deg/s can be interpreted as quenching. Note that only quenching can provide reliable molecular compositions of fluids at the *P–T* conditions of the experiments.

### Kinetics of C-O-H-N fluid equilibration

**Figure 1:**
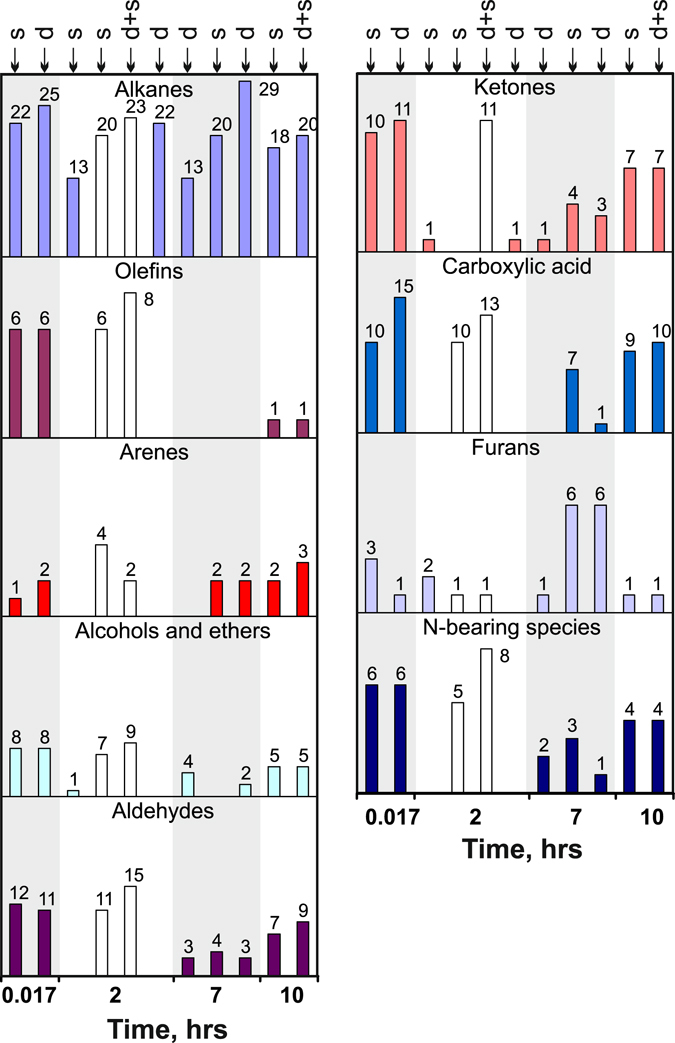
Diversity of alkanes, alkenes, and oxygenated hydrocarbons revealed by GC-MS analysis in quenched C-O-H-N fluids at 6.3 GPa, 1400 °C, MMO-buffered *f*H_2_ and run durations from 0.017 to 10 hours. Symbols on top show starting compositions of charges for fluid generation: d = docosane; d + s = docosane + stearic acid mixture; s = stearic acid. White columns are unquenched samples cooled down at 1 deg/s. Grey and white bands show run groups.

**Figure 2:**
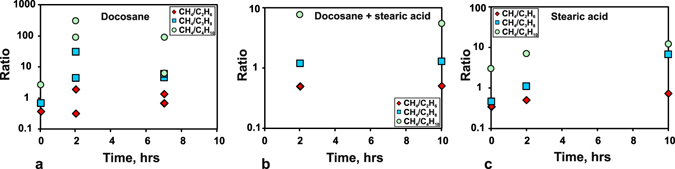
Normalised peak area ratios of CH_4_/C_2_H_6_, CH_4_/C_3_H_8_ and CH_4_/C_4_H_10_ as a function of duration, according to GC-MS analysis of quenched fluids synthesised at 6.3 GPa and 1400 °C from different starting compositions: a = docosane; b = docosane + stearic acid mixture; c = stearic acid.

The effect of kinetics on concentrations of species that form in the C-O-H-N system at 6.3 GPa and 1400 °С was studied in quenching experiments of different durations from 1 min to 10 hours (Fig. 1 and Table 2). The diversity of alkanes revealed by the GC-MS analysis after capsule opening showed almost no run duration dependence. Lower alkanes, which are the dominant carbon species in the C-O-H-N fluids at 1400 °С, did not change much the CH_4_/C_2_H_6_, CH_4_/C_3_H_8_ and CH_4_/C_4_H_10_ ratios of normalised peak areas at longer durations, irrespective of the starting charge composition (Fig. 2). They were slightly higher in fluids generated in longer runs from samples with docosane or stearic acid and remained almost the same in the case of the docosane + stearic acid mixture: the CH_4_/C_2_H_6_ ratio was about 1 in all cases and CH_4_/C_3_H_8_ varied from 1 to 10. Note that CH_4_/C_4_H_10_ reached or slightly exceeded 100 in fluids obtained in 2- and 7-hr long runs from samples with docosane at low H_2_O concentrations but remained below 10 in the case of relatively high H_2_O concentrations with docosane + stearic acid mixture and stearic acid as starting materials.

Olefins, arenes, alcohols, aldehydes, ketones, and carboxylic acids, as well as N-bearing spices, were more diverse in the shortest 1-min runs. Note that the species diversity of oxygenated hydrocarbons was notably smaller in 2-hr experiments but greater in the longest runs (10 hr), though remained below the level of the 1-min run (Fig. 1; 2-hr runs at slow cooling (white histograms) are shown for comparison). Thus the quenched non-equilibrium C-O-H-N fluids obtained in short runs had strongly variable concentrations and high diversity of olefins, arenes, alcohols and ethers, aldehydes, ketones and carboxylic acids, as well as nitrogen-bearing species. The behaviour of these components can be used as evidence of a non-equilibrium composition of reduced fluids.

At 1100 °C, the concentrations of main alkanes and the diversity of oxygenated hydrocarbons in C-O-H-N fluids differed notably in the shortest (1-min) and 7-hour long runs (Tables 2–4). Note that methane was very low while ethane was high (CH_4_/C_2_H_6_ = 0.14) in the shortest run, but their concentrations became similar (CH_4_/C_2_H_6_ = 1.14) and commensurate with those at 1400 °C at the 7 hr duration. The quenched fluids obtained in 7-hr runs at 1100 °C almost lacked oxygenated hydrocarbons, except for some alcohols and ethers and furans. This data shows that the C-O-H-N fluids resulting from thermal decomposition of higher alkanes at 6.3 GPa and 1400 °C can attain equilibrium already in 2-hr runs, while the attainment of equilibrium at 1100 °C most likely requires at least 7-hr durations. Interestingly, alkanes reached equilibrium concentrations in 1 min at 1400 °C, while fluids synthesised in 1-min long runs at 1100 °C were very rich in ethane.

**Table 4:** Representative analyses of quenched reduced C-O-H-N fluids (rel.%) obtained in runs at 6.3 GPa and 1100–1400 °C.

Run^#^		1761_2_3**	1751_2_3	1780_2_2	1727_2_2	889_7_1
Capsule		Pt	Au	Pt	Pt	Pt
Temperature (°C)		1100	1200	1300	1400	1400
Duration (hr)		0.017	7	7	7	7
Starting composition: Docosane^*^						
Water		10.4	9.5	4.2	11	0.7
Alkanes	CH_4_	10.0	49.7	26.9	22.3	51.0
	C_2_H_6_	73.8	31.1	36.5	33.9	38.5
	C_3_H_8_	5.2	8.1	23.9	4.9	8.7
	C_4_H_10_	0.2	1.1	5.7	3.5	0.6
	C_5_H_12_	0.01	0.05	0.2	1.5	0.001
	C_6_-C_15_	≤0.002	—	—	≤0.6	≤0.01
	C_15_-C_19_^***^	≤0.004	—	≤0.4	≤0.2	≤0.01
Olefins		≤0.001	—		—	—
Arenes		≤0.001	—	—	≤0.6	—
Alcohols and ethers		≤0.006	≤0.05	≤0.1	≤0.06	≤0.2
Aldehydes		—	—	—	≤0.3	≤0.001
Ketones		—	—	—	≤0.6	≤0.002
Carboxylic acid		≤0.002	—	—	≤0.2	—
Furans		≤0.001	—	—	—	—
Nitrogen species						
N_2_		0.01	0.02	0.1	0.3	0.02
CH_3_N		>1	>1	>1	0.7	0.05

*All samples contained graphite; **Data given for comparison. ***The quoted concentrations are maximum for a species of the respective group. All GC-MS data are given in Supplementary Table 1.

### Speciation of fluids at *f*O_2_ near IW

**Figure 3:**
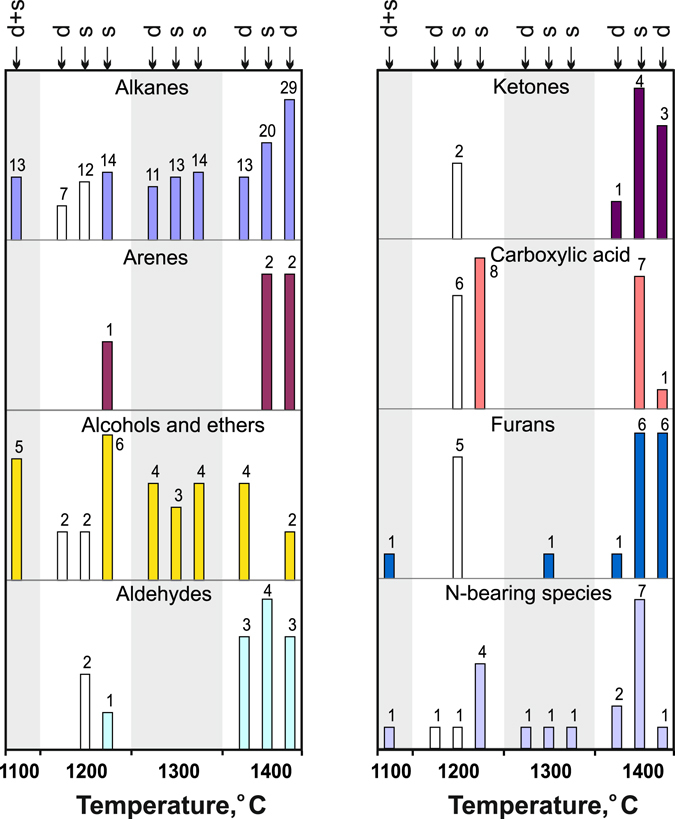
Diversity of alkanes, alkenes, and oxygenated hydrocarbons revealed by GC-MS analysis in quenched C-O-H-N fluids at 6.3 GPa, 1100–1400 °C, MMO-buffered *f*H_2_ and run durations ≥ 2 hours. White columns are samples obtained in Au capsules. Letter symbols are same as in Fig. 2.

**Figure 4:**
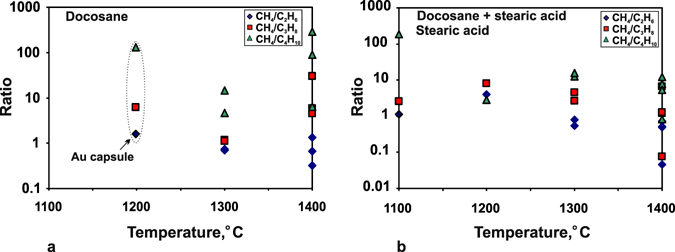
Normalised peak area ratios of CH_4_/C_2_H_6_, CH_4_/C_3_H_8_ and CH_4_/C_4_H_10_ as a function of temperature, according to GC-MS analysis of quenched fluids synthesised at 6.3 GPa from different starting compositions: a = docosane; b = docosane + stearic acid mixture and stearic acid.

We have compared the compositions of carbon and nitrogen species in equilibrated C-O-H-N fluids generated by thermal destruction of docosane in 7-hr long runs at 1100–1200 °C and in ≥ 2-hr runs at 1300–1400 °C (Tables 2 and 4; Figs 3 and 4a), at H_2_O from <1 to 11.2 rel.% (CO_2_ within 0.006 rel.%; CO not detected). Note that the chosen *f*H_2_ buffering technique provided Fe^3+^ to Fe^0^ reduction in a 10-hr test run (Supplementary materials, Figs 1–3). At a higher temperature of 1400 °C, the number of detected alkanes increased from 7–11 at 1200–1300 °C to 14 and even reached 29 in one run. The methane-to-ethane ratios (normalised peak areas) varied from slightly above to slightly below 1, while the CH_4_/C_3_H_8_ and CH_4_/C_4_H_10_ ratios were 1 to 30 and ~100, respectively, without any distinct trend (Fig. 4a). Other species were found in minor amounts of <1 rel.%, except for 0.2 rel.% C_16_-C_17_ alkanes and 0.4 rel.% C_18_ in one run at 1300 °C (Table 4, Supplementary Table 1) against ≤0.01 rel.% in all other runs. Aldehydes and ketones likewise were more diverse in higher-temperature runs at 1400 °C (Fig. 3; white histograms are species in Au capsules), while the number of alcohols and ethers remained approximately the same.

As for nitrogen speciation, the experimental results (Tables 2, 4, Supplementary Table 1) showed methanimine (CH_3_N, CAS # 2053-29-4) to be the predominant nitrogen host in N-poor C-O-H-N fluids. However, N_2_ was persistent, though minor (0.1 to 0.01 rel.%), even in ultra-reduced C-O-H-N fluids resulting from decomposition of docosane, with the CH_3_N/N_2_ > 2 ratio in all cases. Ammonia appeared in a single 1-min run at 1400 °С, with its concentration notably exceeding both N_2_ and CH_3_N. Other nitrogen species were detectable in trace amounts (Fig. 3 and Supplementary Table 1).

### Speciation of fluids at *f*O_2_ slightly above IW

**Figure 5:**
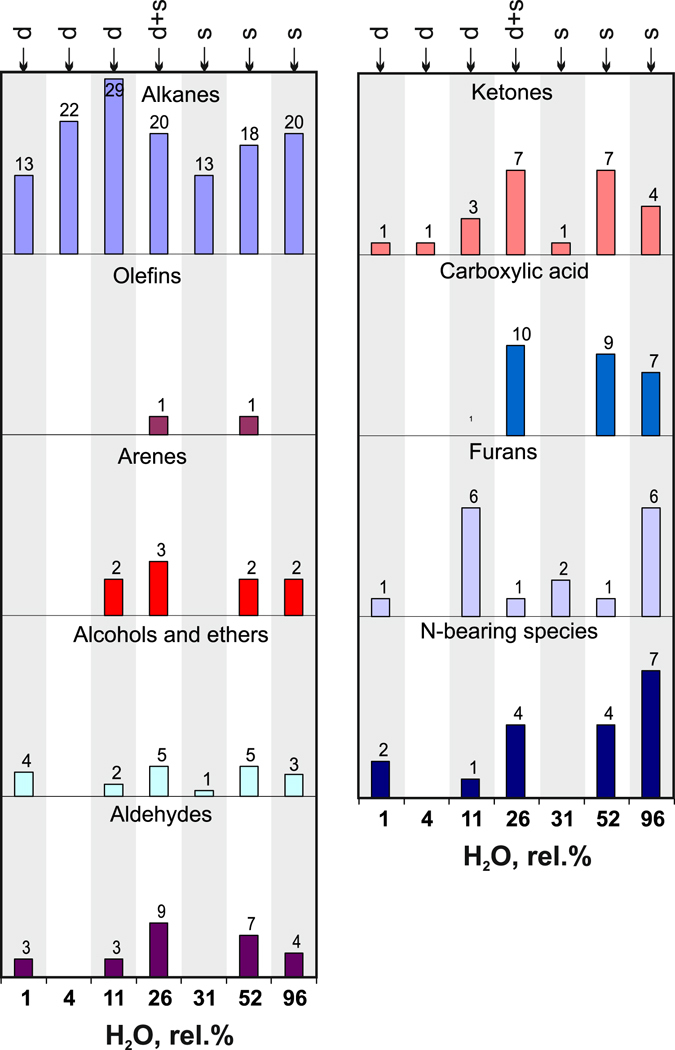
Diversity of alkanes, alkenes, and oxygenated hydrocarbons revealed by GC-MS analysis in quenched C-O-H-N fluids as a function of normalised areas of H_2_O at 6.3 GPa, 1400 °C, run duration ≥2 hours in MMO-buffered *f*H_2_ and in unbuffered experiments. Letter symbols are same as in Fig. 2.

The normalised peak areas of water in the chromatograms of quenched equilibrium fluids generated by thermal decomposition of stearic acid or the stearic acid + docosane mixture varied from 16 to 55 rel.%. The concentration of CO_2_ reached 0.9 rel.% at 1100 °С but decreased to 0.01–0.1 rel.% at 1300–1400 °С. No CO was detectable at higher water contents, as in the case of more reduced fluids. C_1_-C_4_ alkanes in C-O-H-N fluids showed more prominent trends with increasing water contents (Figs 2–5). As the run temperature increased to 1400 °С instead of 1200 °С, the CH_4_/C_2_H_6_ ratio of normalised peak areas decreased only from slightly above 1 to slightly below 1, as at low H_2_O, but the CH_4_/C_3_H_8_ and CH_4_/C_4_H_10_ ratios became ten times smaller: from 10 to 1 and from 100 to 10, respectively. Note that CH_4_/C_4_H_10_ at 1400 °С were much lower at high than at low water contents.

Synthesis in Pt capsules at 1100  and 1300 °С gave fluids with C_16_-C_19_ alkanes from 0.2 to 0.9 rel.% and to 0.2 rel.%, respectively (Table 5). The respective alkane contents in fluids from Au capsules at 1200 °С were no higher than 0.06 rel.% and were still lower in Pt capsules at 1400 °С. Oxygenated hydrocarbons in the fluids were diverse in both Au and Pt capsules (Fig. 3). The contents of the detected species did not show marked variation trends. Among oxygenated hydrocarbons, alcohols and carboxylic acids had the largest contents. Note that a water-rich quenched fluid synthesised at 1300 °С in a 7-hr long run contained 0.1 rel.% methanol, ethanol, and benzoic acid (C_7_H_6_O_2_).

**Table 5:** Representative analyses of quenched H_2_O-rich C-O-H-N fluids (rel.%) obtained in runs at 6.3 GPa and 1100–1400 °C.

Run^#^		1769_2_2	1751_2_2	1780_2_3	1019_7_3	1019_7_1
Capsule		Pt	Au	Pt	Pt	Pt
Temperature (°C)		1100	1200	1300	1400	1400
Duration (hr)		7	7	7	10	10
Starting composition*		Stearic acid + docosane	Stearic acid	Stearic acid	Stearic acid + docosane	Stearic acid
Water		16.4	40.6	54.6	26.5	51.9
Alkanes	CH_4_	35.2	37.2	13.1	17.9	17.9
	C_2_H_6_	30.8	18.2	24.0	35.3	24.6
	C_3_H_8_	13.6	2.7	4.9	13.9	2.7
	C_4_H_10_	0.2	0.9	0.9	3.3	1.5
	C_5_H_12_	0.02	0.05	0.03	0.2	0.1
	C_6_-C_15_	≤0.04	≤0.001	≤0.1	≤0.04	≤0.02
	C_15_–C_19_^**^	≤0.9	≤0.06	≤0.2	≤0.02	≤0.01
Olefins	—	—	—	≤0.001	≤0.01	≤0.01
Arenes	—	—	—	≤0.003	≤0.001	≤0.001
Alcohols and ethers	≤0.2	≤0.002	≤0.8	≤0.01	≤0.02	≤0.02
Aldehydes	—	≤0.05	—	≤0.003	≤0.02	≤0.02
Ketones	—	≤0.01	—	≤0.007	≤0.01	≤0.01
Carboxylic acid	—	<0.001	≤0.1	≤0.09	≤0.02	≤0.02
Furans	≤0.006	≤0.001	≤0.003	≤0.001	≤0.01	≤0.01
Nitrogen species						
N_2_	0.4	0.02	0.08	0.01	0.01	0.01
CH_3_N	0.1	>1	—	>1	0.9	0.9

*All samples contained graphite. **The quoted concentrations are maximum for a species of the respective group. All GC-MS data are given in Supplementary Table 1.

**Figure 6:**
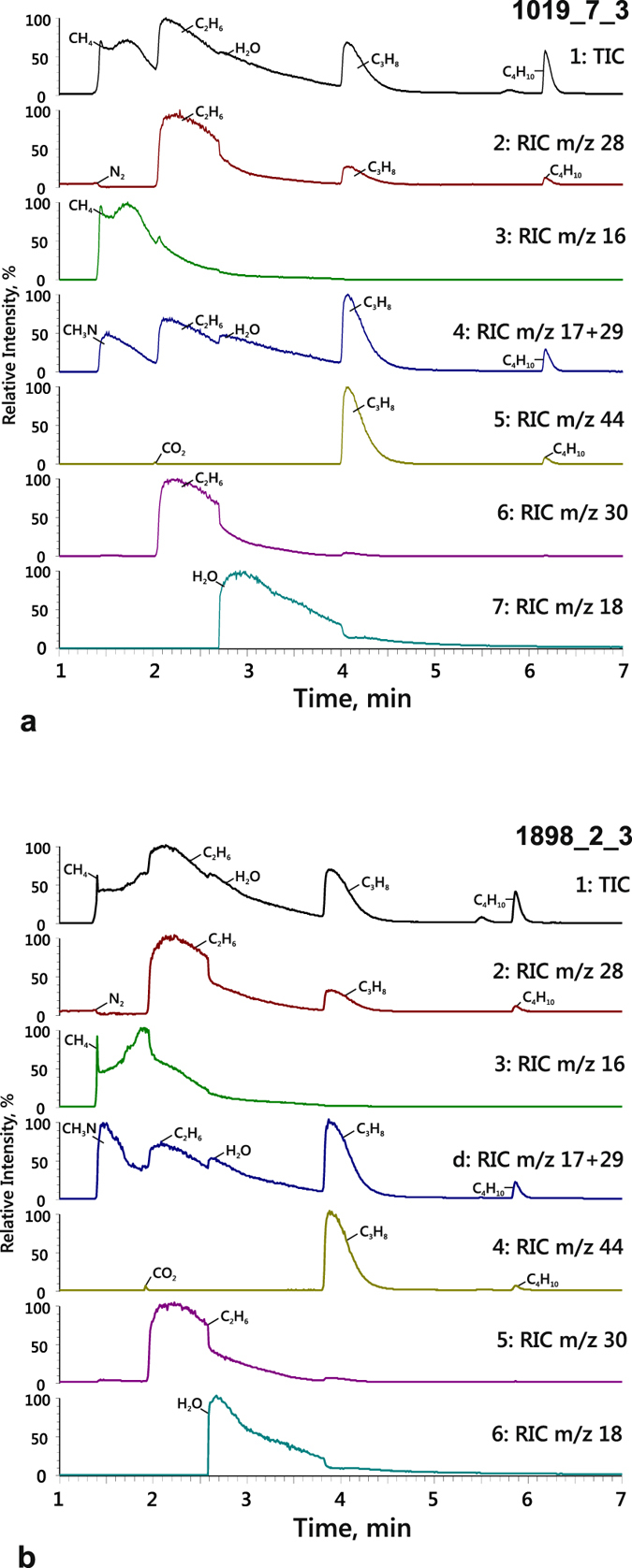
Fragments of chromatograms of quenched fluids extracted from Pt capsules after runs 1019_7_3 (**a**) and 1898_2_3 (**b**). 1 = Total ion current (TIC) chromatograms; 2 = Reconstructed ion chromatograms (RIC) m/z 28 that characterise traces of molecular nitrogen, ethane, propane and butane; 3 = RIC m/z 16: methane; 4 = RIC m/z 17 + 29: methanimine; 5 = RIC m/z 44: traces of carbon dioxide, propane and butane; 6 = RIC m/z 30: ethane; 7 = RIC m/z 18: water.

Methanimine (CH_3_N) was the main nitrogen species of C-O-H-N fluids at high-H_2_O as well (Table 2, Fig. 6 and Supplementary Table 1), but no ammonia was detected in any run. Persistent N_2_, from 0.02 to 0.4 rel.%, was observed in the fluids generated by decomposition of stearic acid or the stearic acid + docosane mixture, as in the case of reduced fluids. The normalised peak area ratios corresponding to CH_3_N/N_2_ in quenched fluids were A(17 + 29 m/z)/A(28 m/z) ≫ 1 in most of analysed spectra. Methanimine predominated in the fluids from both Au and Pt capsules. It was found in small amounts only in four runs with Pt capsules (out of those, *f*H_2_ was not buffered in two runs and little fluid was released upon capsule opening in one run). Note that the concentration of CH_3_N increased at slow cooling (Fig. 6a,b).

### Composition of fluids near CW

Oxygen fugacity of C-O-H-N fluids could reach the water maximum (CW) in two unbuffered runs at 1200 and 1400 °С (Table 6) because of hydrogen leakage^30, 31^. The loss of hydrogen from the fluid led to its water enrichment and oxidation of the hydrocarbons originally produced by thermal decomposition of the starting materials. Contrary to our expectations, HCs demonstrated different degrees of stability to oxidation. The concentrations of methane and ethane decreased dramatically from 20–30% to a few percent and even to fractions of percent (Table 6) while the C_3_, C_4_ and C_5_ alkanes remained almost invariable. Some higher alkanes, especially, C_16_, were present in notable amounts: 0.04 rel.% at 1200 °С to 0.2 rel.% at 1400 °С. In the lower-temperature runs phtalates were observed as the main species among oxygenated hydrocarbons, possibly as a result of capsule contamination. The detected species were markedly more diverse in higher-temperature runs, where acetic acid was predominant. The diversity of HC species in fluids with 1 to 96 rel.% H_2_O (normalised peak areas) exhibited no distinct trends (Fig. 5). The fluids near CW contained N_2_ as the predominant nitrogen species and trace amounts of nitriles and azines (Supplementary Table 1).

**Table 6:** Representative analyses of quenched H_2_O-rich C-O-H-N fluids (rel.%) obtained in unbuffered runs at 6.3 GPa.

Run^#^		1315_3_5	1720_2_2
Capsule		Pt	Pt
Temperature (°C)		1200	1400
Duration (hr)		7	7
Starting composition*		Stearic acid	Stearic acid
Water		93.5	96.3
Alkanes	CH_4_	2.9	0.06
	C_2_H_6_	0.7	1.4
	C_3_H_8_	0.4	0.08
	C_4_H_10_	1.0	0.8
	C_5_H_12_	0.02	0.1
	C_6_-C_14_^**^	—	≤0.08
	C_15_-C_19_	≤0.04	≤0.2
Olefins		—	—
Arenes		—	≤0.008
Alcohols and ethers		≤0.7	≤0.01
Aldehydes		≤0.003	≤0.01
Ketones		—	≤0.01
Carboxylic acid		≤0.008	<0.07
Furans		≤0.006	≤0.01
Nitrogen species			
N_2_		0.5	0.01
CH_3_N		—	—

*All samples contained graphite. **The quoted concentrations are maximum for a species of the respective group. All GC-MS data are given in Supplementary Table.

### Calculated *f*O_2_ and carbon contents

**Figure 7:**
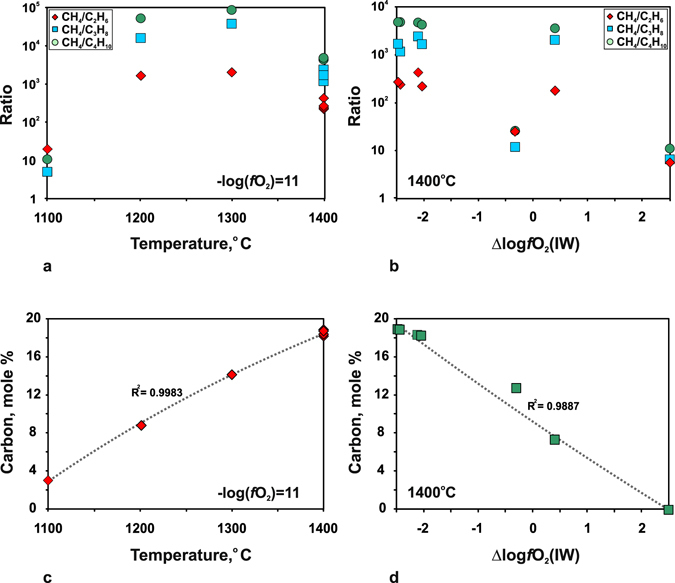
Composition of quenched fluids converted using GC-MS calibration with respect to main species: CH_4_, C_2_H_6_, C_3_H_8_, C_4_H_10_ and H_2_O. a: mole ratios CH_4_/C_2_H_6_, CH_4_/C_3_H_8_ and CH_4_/C_4_H_10_ in strongly reduced fluids at 6.3 GPa, 1100–1400 °C and constant *f*O_2_; b: same at 6.3 GPa, 1400 °C in a range of *f*O_2_; c: amount of carbon in fluids as a function of temperature; d: amount of carbon in fluids as a function of *f*O_2_.

Oxygen fugacity (*f*O_2_) is the key parameter of fluid systems against which to compare the compositions of fluids synthesised in quenching experiments with data on natural mantle fluids. *f*O_2_ in the experimental samples was calculated by Gibbs energy minimisation at 6.3 GPa and 1100–1400 °C knowing the components of the obtained fluids. However, chromatography–mass spectrometry revealed more than 30 carbon and nitrogen species in the fluids, and the calculations required some assumptions. Primarily, the presence of nitrogen species found in very low concentrations was neglected, i.e., the C-O-H-N system was reduced to the C-O-H one. The contribution of alkanes higher than C_5_, as well as oxygenated hydrocarbons and CO_2_, all below 1 rel.%, into the *f*O_2_ of the system was likewise assumed negligible. With these assumptions, the system composition was simplified to H_2_O, CH_4_, C_2_H_6_, C_3_H_8_, and C_4_H_10_, and this limited number of main species was used in CG-MS calibrations (see Supplementary information). The normalised peak areas of particular components were converted to mole % only for a part of quenching experiments where equilibrium concentrations of the main components were obtained. They were further used to estimate C contents in the fluids as a function of temperature at constant *f*O_2_ and as a function of *f*O_2_ at a constant temperature of 1400 °C (Table 7 and Fig. 7). The results generally record markedly lower carbon contents at lower temperatures and higher *f*O_2_.

**Table 7:** Mole ratios of CH_4_, C_2_H_6_, C_3_H_8_ and C_4_H_10_, carbon content (mole %), and calculated *f*O_2_ in fluid phase, according to GC-MS calibration (see text for explanation).

Run^#^	Capsule	Buffer *f*H_2_	T, (°C)	Calc. *f*O_2_	CH_4_/C_2_H_6_	CH_4_/C_3_H_8_	CH_4_/C_4_H_10_	C, mole %
1769_2_2	Pt	MMO	1100	−10.8	20	5.1	10.8	3.0
1315_3_5	Pt	—	1200	−8.5	227	1384	839	0.01
1751_2_2	Au	MMO	1200	−10.8	2012	14912	47973	8.7
1751_2_3	Pt	MMO	1200	−15.4	2774	18853	59694	19.9
1746_2_3	Pt	MMO	1300	−9.0	22	109	237	6.3
1746_2_2	Pt	MMO	1300	−10.5	2063	36898	85957	14.1
1780_2_2	Pt	MMO	1300	−9.3	6.9	8.7	17	8.9
1780_2_3	Pt	MMO	1300	−8.5	5.7	2.8	5.8	4.9
1753_2_3	Pt	MMO	1400	−10.6	433	2412	4733	18.3
1898_2_1	Pt	MMO	1400	−10.5	224	1677	4297	18.2
1898_2_3	Pt	MMO	1400	−10.9	244	1181	4804	18.8
1720_2_2	Pt	—	1400	−6.0	5.7	6.7	11.1	0.01
1727_2_2	Pt	MMO	1400	−8.8	26	12	24.98	12.6
1019_7_1	Pt	MMO	1400	−8.1	176	2016	3471	7.3
1019_7_3	Pt	MMO	1400	−11.0	274	1711	4797	18.9

## Discussion

### Processes of fluid equilibration

**Figure 8:**
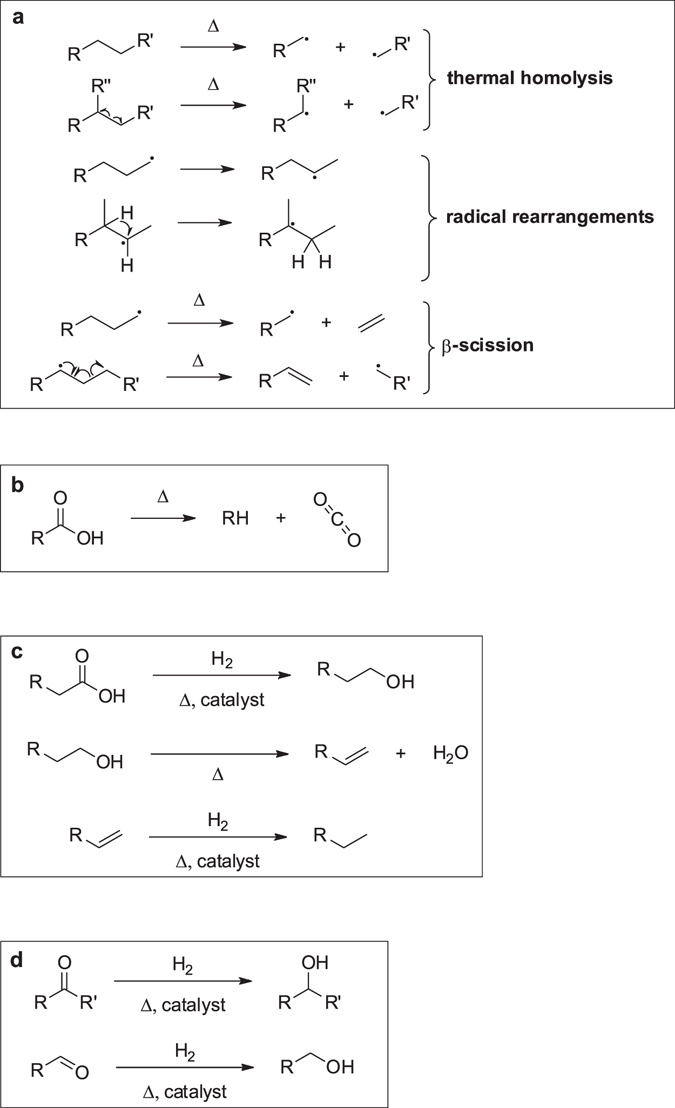
Main chemical processes during formation of equilibrium fluids. (**a**) initial thermal formation of radical species from higher hydrocarbons in homolysis, rearrangement,  their further rearrangement and β-scission reactions; (**b**) thermal decarboxylation of carboxylic acids; (**c**) processes involving carboxyl acids; (**d**) hydrogen reduction of carbonyl compounds.

According to the reported results, the fluids experimentally generated at 6.3 GPa and 1100–1400 °C attain equilibrium with respect to all C-O-H-N components for the time from 2 to 7 hours. The greatest diversity of carbon species observed in 1-min runs indicates that thermal decomposition of docosane and stearic acid produces numerous non-equilibrium components of the system which disappear in longer runs. GC-MS analysis reveals light alkanes as main run products, with predominant methane and ethane (Table 2). The changes the HCs experience at the experimental conditions can either lengthen or shorten the hydrocarbon chains. For higher hydrocarbons, decomposition most likely occurs as thermal cracking^35^ and appears to be the main process involving n-alkanes, including n-docosane and alkyl chains of fatty acids. In principle, the initial thermal formation of radical species from higher hydrocarbons in homolysis and rearrangement reactions leads to further β-scission into alkene and alkyl radicals with shorter chains (Fig. 8a). Ethene that forms in β-scission of terminal radicals in excess of H_2_ readily reduces to ethane. The radicals resulting from cracking can recombine or react with alkanes or alkenes to form alkanes and new radicals.

Thermal decarboxylation (Fig. 8b) of carboxylic acids (such as stearic acid) produces alkanes shorter for one carbon atom (e.g., relatively high amounts of heptadecane (Supplementary Table 1) in run #1016_7_2) which decompose in a regular way through dehydrogenation and β-scission (Fig. 8a). Catalytic reduction of fatty acids in excess of hydrogen yields primary alcohols. Further dehydration of alcohols to alkenes may be followed by catalytic hydrogenation on the Pt surface producing a mixture of alkanes and terminal alkenes (Fig. 8c).

However, radical reactions of intermediates are also possible at each step of reduction, and varieties of shortened primary alcohols were observed in the experiments. The reactions can be catalysed by Pt, judging by the presence of methanol and ethanol in the fluids from Pt capsules and their absence in the case of Au capsules in 1200 °C runs # 1315_3_5, 1751_2_2 and 1751_2_3 (Supplementary Table 1).

The fluids generated in Au and Pt capsules differ in carbon speciation irrespective of run duration and temperature: methane is one of major fluid components (along with water) in both cases but the methane/ethane ratios of normalised peak areas are, respectively, CH_4_/C_2_H_6_ > 1 and <0.8 in the Au and Pt capsules (Table 2). This difference may be due to catalytic processes that involve Pt, which may facilitate homolytic dissociation of methane to methyl radicals on the surface at temperatures over 1000 °C^36^. The process accelerates with heating, and the methane/ethane ratio may decrease at higher temperatures. Indeed, the CH_4_/C_2_H_6_, CH_4_/C_3_H_8_, and CH_4_/C_4_H_10_ mole ratios we obtained by calibration were the lowest at 1400 °C and at constant *f*O_2_ (Fig. 7a). The 7-hr run at 1100 °C gave a spike, possibly, because the system failed to attain equilibrium. Further recombination of the formed radicals produces a mixture of lower HCs: ethane, propane and *n*-butane. The reactivity of ethane is restricted to catalytic dehydrogenation on the Pt surface with formation of ethene, but the excess of hydrogen (*f*H_2_ buffered at MMO) impedes this process by shifting the equilibrium toward the reagents. For this reason, ethane does not contribute to the formation of methyl/ethyl radicals and, hence, methane and higher HCs, and tends to accumulate. Nevertheless, the catalytic fission of *n*-butane produces two ethyl radicals that recombine and react with alkenes with further formation of higher HCs.

The fluids generated in both Au and Pt capsules contained various oxygen-bearing compounds, mainly alcohols, ethers, aldehydes, ketones and furans (Tables 4, 5, Supplementary Table 1 and Fig. 3). However, the contents of carbonyl compounds (aldehydes and ketones) were lower in less reduced fluids from Pt samples (Table 5), at comparable T-τ conditions, possibly, because of catalytic hydrogen reduction (Fig. 8d) on the Pt surface and subsequent dehydration of alcohols. The run duration dependence of carbon speciation patterns in Pt capsules (Fig. 1) shows that aldehydes and ketones are the least diverse in the case of 2-hr runs but more diverse in fluids generated in 10-hr runs, possibly, because the catalytical activity of Pt decreases with time.

### Carbon speciation

Main carbon species in the synthesised equilibrium fluids are CH_4_, C_2_H_6_, C_3_H_8_ and C_4_H_10_, and their concentrations (according to GC-MS analysis) agree well with the calculated relations of CH_4_ > C_2_H_6_ > C_3_H_8_ > C_4_H_10_^20, 25, 28, 32, 33^ almost in all cases (Fig. 7а,b and Table 7). The CH_4_/C_2_H_6_, CH_4_/C_3_H_8_, and CH_4_/C_4_H_10_ ratios are lower in the case of both higher temperature (1400 °С) and *f*O_2_ (2.5 log units above IW) at 6.3 GPa (Fig. 7а,b). Taking into account the H_2_O variations, this dependence is the reason why temperature and *f*O_2_ control the amount of carbon the hydrocarbon fluids can carry (Fig. 7c,d). Previous calculations by Huizenga *et al*. (ref. 25) for 5.0 GPa and 1227 °C gave a similar *f*O_2_ dependence of carbon contents in fluids synthesised in the C-O-H system. This similarity indicates that thermodynamic calculations provide a faithful idea of C behaviour in fluids subject to oxidation at CLM pressures. On the other hand, as we have demonstrated experimentally, the amount of carbon in the fluid phase can be notably lower at a lower temperature (1100 °C) at a constant oxygen fugacity of −log(*f*O_2_) = 11.

The equilibrium compositions of the GC-MS analysed C-O-H-N fluids count more than 30 carbon and nitrogen species and contain up to 1 rel.% С_15_-С_18_ alkanes, alcohols, aldehydes, ketones, carboxyl acids, and furans (Tables 4–6 and Figs 1, 3 and 5). In some cases, the contents of species in the obtained fluid vary largely even in runs of the same temperature and starting composition. This variation may result from the presence of moisture that adsorbs, in smaller or larger amounts, on graphite flakes during the capsule assembly. The reduced fluids at *f*O_2_ near IW generated by thermal decomposition of docosane are relatively rich only in alcohols, and their composition apparently corresponds to *f*O_2_ at which diamonds crystallise from metal melts (ref. 12). At higher H_2_O, the concentrations of С_15_-С_18_ alkanes increase slightly, while the diversity remains basically the same in alkanes and increases notably in oxygenated hydrocarbons (especially, ketones and carboxylic acids). The effect of lower temperature (1200 °C instead of 1400 °C) is to reduce the diversity of alkanes and oxygenated hydrocarbons in more reduced fluids. As H_2_O exceeds 90 mole % and *f*O_2_ reaches 2.5 log units above IW, methane in the fluid almost zeroes down, while higher alkanes do not change much (Tables 4–6 and Fig. 5). For instance, the fluids with *f*O_2_ near CW contain 1 rel.% C_2_H_6_, fractions of percent C_3_H_8_ and C_4_H_10_, and C_15–19_ alkanes, as well as quite large amounts of oxygenated hydrocarbons, especially alcohols and carboxylic acids.

The effect of Pt and Au capsules on the composition of equilibrium C-O-H-N fluids is seen in the results of 7-hr long runs at 1100–1300 °C (Table 2 and Fig. 3) where CH_4_/C_2_H_6_ in the fluids from Au capsules are higher than with Pt capsules (Table 2) which become involved in catalytic processes (see above). Fluids generated in the two types of capsules are similar in diversity of alkanes (Fig. 4), while H_2_O-rich fluids from Pt capsules have low or absent aldehydes and ketones, possibly, due to catalytic hydrogen reduction at the Pt surface, and subsequent dehydration of the formed alcohols. The composition similarity of the quenched fluids synthesised in Pt and Au capsules indicates that the Pt effect consists in faster attainment of equilibrium in the system and must be the smallest in 10-hr runs.

All detected carbon species, except alkanes, turn out to be very sensitive to cooling rates: they are much more diverse at slow (1 deg/s) cooling (Fig. 1). Therefore, back reactions upon cooling lead primarily to synthesis of oxygenated hydrocarbons.

### Nitrogen speciation

**Figure 9:**
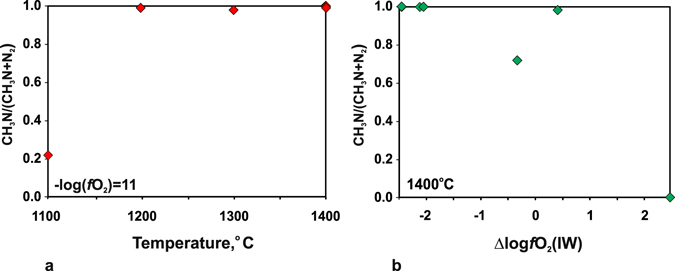
Normalised peak area ratios of CH_3_N and N_2_ that characterise their role as nitrogen species in C-O-H-N fluids in a large range of *T*-*f*O_2_ parameters at 6.3 GPa. (**a**) 1100–1400 °C and constant *f*O_2_; (**b**) redox interval from strongly to moderately reduced conditions.

**Figure 10:**
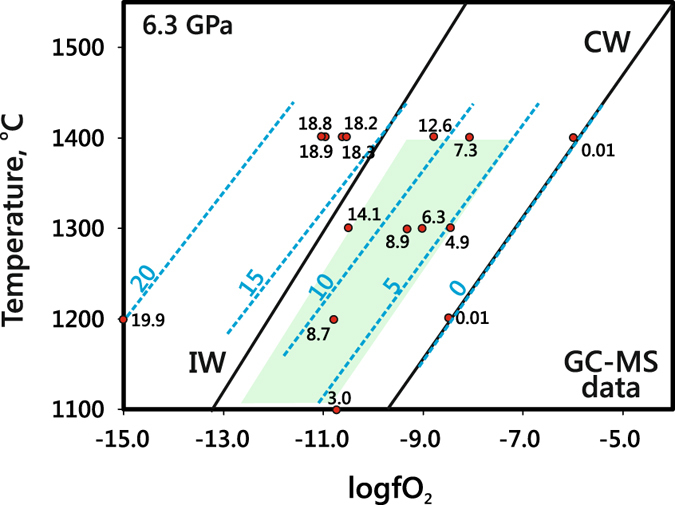
Concentrations of carbon (mole %) in reduced C-O-H-N fluids at 6.3 GPa, according to GC-MS analysis. Red circles are GC-MS-measured values for experimental compositions; blue dash line is the carbon isopleth. Light-green field shows T-*f*O_2_ range for cratonic lithospheric mantle at a depth of ~200 km (see text for explanation). IW and CW curves are after refs 1, 24 and 56.

Nitrogen in the studied C-O-H-N fluids is mainly air N_2_ entrapped by the capsule assembly, which reacts with hydrogen, hydrocarbons, and graphite at the experiment *P-T* conditions to form methanimine (Table 2, Figs 6 and 8): CH_3_N with the formula . The fluids also contain trace amounts of other nitrogen species, such as some nitriles, mainly at 1400 °C and high H_2_O contents. CH_3_N/N_2_ ratios estimated by GC-MS (Fig. 9) show that CH_3_N is the main nitrogen species in almost all equilibrium reduced fluids; CH_3_N/N_2_ < 1 only at 1100 °C and *f*O_2_ about −11 log units. However, CH_3_N disappears at 1400 °C when *f*O_2_ becomes four orders of magnitude higher and approaches CW. The temperature and redox dependence of CH_3_N stability (with regard to the slope of buffers in the T-*f*O_2_ diagram of Fig. 10) indicates that the formation of CH_3_N is possible at low *f*O_2_.

Thus, CH_3_N discovered in the C-O-H-N system at 6.3 GPa, 1100–1400 °C, and a fluid C/N ratio of ∼20 may be an important component in reduced mantle containing minor amounts of nitrogen. It may predominate as a nitrogen host already at *f*O_2_ near and below IW. A more detailed study is obviously required to constrain the stability of CH_3_N at the mantle *P-T-f*O_2_ conditions, with a special focus on nitrogen sources in the reactions that produce this species. Note that NH_3_ and N_2_ are stable in the N-rich C-O-H-N system generated by decomposition of melamine, as well as its mixture with stearic acid or docosane at C/N ratio in the fluid from 0.4 to 4.6, at similar *P-T*-*f*O_2_^34^, while the redox stability of CH_3_N is comparable with that of ammonia^3, 34^. To sum up, the behaviour of CH_3_N may control the deep nitrogen cycle in N-poor reduced peridotitic mantle. Specifically, silicate phases capable of dissolving ammonia in the presence of NH_3_-bearing fluids^2, 19, 37^ hardly can host nitrogen in equilibrium with CH_3_N-bearing mantle fluids.

### Carbon and nitrogen transport across redox and thermal barriers in the mantle

**Figure 11:**
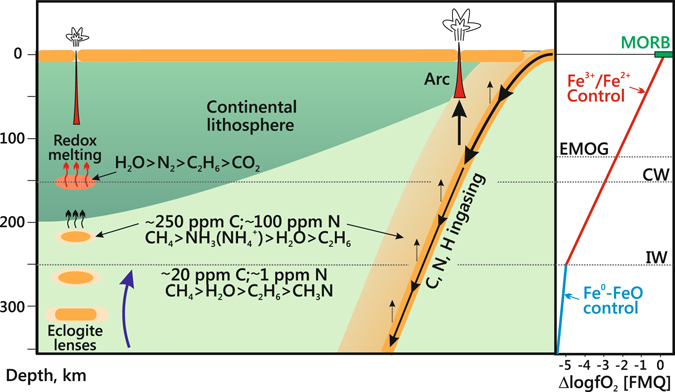
Schematic of the upper mantle volatile cycle and main carbon and nitrogen species of C-O-H-N fluids in depleted and enriched domains^38, 39^. Black arrows show paths and estimates of the relative magnitudes of carbon, nitrogen and hydrogen fluxes and blue arrow is mantle upwelling. Right panel: potential mantle *f*O_2_ as a function of depth. The Fe^0^-FeO and Fe^3+^/Fe^2+^ (equilibria involving the exchange of Fe^3+^ and Fe^2+^ between silicate minerals) curves are according to refs 1 and 56. Oxygen fugacity of CW (maximum H_2_O content in C-O-H fluids), EMOD/G (equilibria enstatite + magnesite = olivine + graphite/diamond) and MORB (mid-ocean ridge basalts) are according to refs 1 and 24. ‘Redox melting’ process at depths of 120–150 km is according to refs 1 and 24.

The most important carbon and nitrogen species of C-O-H-N fluids in the upper mantle revealed in this study of N-poor fluids, as well as in previous results for N-rich fluids^34^, are CH_4_ and CH_3_N in the depleted domains with ∼20 ppm C and ∼1 ppm N^38, 39^, and CH_4_ and NH_3_ in the enriched domains containing ∼250 ppm C and ∼100 ppm N^38, 39^ (Fig. 11). Note that this inference is based on experiments with a simplified C-O-H-N system limited to four components, whereas the speciation in the natural mantle fluids is much more complex. Namely, N-rich fluids derived from a subducting slab may be quite rich in chlorides^40^. The appearance of the Cl^−^ ion in the fluid may be coupled to NH_4_^+^ forming a stable ligand. Furthermore, the real fluid phase in the mantle occurs in the interstitial space of silicate rocks. As it was shown previously^6^, the pH values of the eclogitic fluids are strongly alkaline, which supports the model of ionic C-bearing species. At the same time, fluids in equilibrium with mantle peridotite minerals generally contain species in the molecular form^6^, which is consistent with the conventional fluid models.

The ascent of N-bearing reduced fluids is an important element of the carbon and nitrogen cycles in the mantle^2, 5^. On their way to shallow mantle, these fluids can cross redox and/or temperature barriers, such as multiple redox fronts^24^ in the lithosphere or the boundary between hotter asthenosphere and colder lithosphere^41^. Our experiments and published calculation results^20, 25, 28, 32, 33^ have implications for the behaviour of fluids crossing such redox and thermal barriers. Carbon concentrations in reduced fluids within the *T*-*f*O_2_ range of CLM at a depth of ∼200 km vary as plotted in Fig. 10. The reduced fluids stable at ∼IW lose almost all carbon (about 18 mole % С, Figs 7e and 10) upon isothermal oxidation at 1400 °С to *f*O_2_ about CW. For the fluids with methane as a predominant carbon species, the experimentally obtained and calculated^25^ for 5.0 GPa and 1227 °C amounts of carbon released during oxidation are in good agreement. The novelty of the experimental results is that HCs show different degrees of stability to oxidation, and this difference can affect the carbon speciation in the fluid. Namely, oxidation can be expected to decrease the concentrations of methane and ethane (much more strongly for the former) but to cause almost no effect on C_3_, C_4_ and C_5_ alkanes (Table 2 and Fig. 7b), as well as on some C_15_-C_19_ alkanes which remain within fractions of percent (Table 6). Note also that H_2_O-rich fluids at 1400 °С contain diverse oxygenated hydrocarbons (Fig. 5), with predominant acetic acid. As for nitrogen species, we infer CH_3_N to become less important and the role of N_2_ to grow as oxidation progresses (Fig. 9b).

The question whether any hydrocarbons can survive transport across the mantle redox fronts is essential for understanding the deep carbon cycle. Neither the stability of higher alkanes at *f*O_2_ near CW nor the oxidation patterns of hydrocarbon fluids have been studied experimentally at mantle *P-T* conditions. In our previous quenching experiments with anthracene (C_14_H_10_), performed without external *f*H_2_ buffering^31^, oxidation of the C-O-H system produced an aqueous fluid with trace amounts of CH_4_ (0.5 to 0.7 mole%) and C_2_H_6_ (≤0.1 mol.%). According to the data of this study, hydrous silicate magmas generated within multiple redox fronts can entrap minor amounts of some higher alkanes and oxygenated hydrocarbons of mantle origin and carry them further to shallow mantle.

As modeling by Stachel and Luth (ref. 5) demonstrates, less than 50 ppm fluid are required to completely reset the redox state of depleted cratonic peridotite to that of the fluid. Taking into account the strongly reduced chemistry of most peridotites at the depths of diamond stability^20–23^, Stachel and Luth (ref. 5) conclude that redox fronts can be unstable to interaction with hydrocarbon fluids and that the last fluids to interact with the deep CLM are generally reducing. The fluids which penetrate into reduced but colder CLM domains and cool down from 1400 °С and *f*O_2_ ∼IW to 1100 °С at *f*O_2_ slightly above IW are inferred to loose carbon (decreasing from 18 to 3 mole %) but gain water (Figs 7a,c and 10). The cooled fluids contain more ethane, propane, and butane but much less diverse oxygenated hydrocarbons, mainly methanol and ethanol (Fig. 3 and Table 5, Supplementary Table 1). As for nitrogen speciation, N_2_ is expected to be predominant instead of CH_3_N (CH_3_N/N_2_ < 1) (Fig. 9a) in the cooled peridotitic fluid, which contains mainly molecular forms of species.

### Diamond formation

Carbon-bearing fluids in CLM can act as both carbon carriers and diamond crystallisation environment^5, 42–45^. The reaction of hydrocarbons with O_2_ at multiple redox fronts^24^ stimulates the activity of H_2_O and causes drastic reduction of total carbon content in the fluids, as well as rapid drop in the solidus temperature of the ambient rocks. The redox melting is considered to be an important process in the cratonic mantle lithosphere^24^. As a result of oxidation, carbon of methane and other hydrocarbons is inferred to release in the form of C^0^ and to become consumed for saturation with the aqueous fluid. Experimental data show that this C^0^ could be involved in diamond crystallisation. The chemistry of mineral inclusions in diamond^46, 47^ and stable isotope data of carbon and nitrogen^48^ indicate distinct possibility of diamond precipitation from CH_4_. Higher alkanes and some oxygenated hydrocarbons have been shown experimentally to be stable against oxidation of hydrocarbon fluids till the water maximum (Table 6). Therefore, they should be present in the diamond crystallisation environment, along with water, and can be entrapped as inclusions. Recent studies^8–11^ confirm the presence of higher HCs in inclusions from diamond and its syngenetic mantle-derived minerals.

The available experimental data on crystallisation of diamond from the fluid phase^49–53^ indicate the existence of important, possibly, kinetic barriers impeding its spontaneous nucleation and further growth in the mantle fluid. Diamond has never been synthesised from strongly reduced fluids in the field of its thermodynamic stability, even in the presence of metastable graphite^31, 52^. Note that none of our experiments, from 1 minute to 10 hours long, led to spontaneous diamond nucleation, even near CW. As we showed earlier^31^, diamond can nucleate and grow at run duration at least 40 hours, at 1600 °C and at relatively high *f*O_2_ near CW, in H_2_O-rich fluids. At lower temperatures, diamond nucleation begins even in oxidised carbonatitic fluids after a much longer induction period^44, 51, 54^. Thus, only oxidation of fluids within redox fronts at temperatures approaching 1400 °С can maintain effective diamond formation. Fluids cooling in strongly reduced mantle can release excess carbon as metastable graphite. The fate of this graphite within CLM may be different: it may either remain in the metastable state for an infinitely long time or convert to diamond upon interaction with oxidised alkaline metasomatic fluids^44, 51, 54^. This mechanism is similar to the model suggested by Jablon and Navon (ref. 55) as universal for most diamonds from CLM.

## Conclusions

Experiments at 6.3 GPa show that fluids generated by thermal decomposition of docosane and stearic acid can attain equilibrium for 2 and 7 hours at 1400 and 1100 °C, respectively. The shortest 1-min runs lead to the formation of numerous non-equilibrium components of the system, especially oxygenated hydrocarbons which disappear in longer runs. The processes leading to equilibrium are mainly radical and include thermal formation of radical species from higher hydrocarbons and carboxylic acids in homolysis, rearrangement reactions, and further β-scission into alkenes and alkyl radicals. Carboxylic acids additionally undergo thermal decarboxylation. Equilibrium fluids contain CH_4_, C_2_H_6_, C_3_H_8_ and C_4_H_10_ as main carbon species, which is consistent with previous experimental and theoretical results^25, 28, 29, 31, 32^. It has been demonstrated for the first time that equilibrium N-poor C-O-H-N fluids can contain more than 30 carbon and nitrogen species. Besides the main species, they include С_15_-С_19_ alkanes, alcohols, aldehydes, ketones, carboxylic acids, and furans.

The carbon and nitrogen speciation in the equilibrium fluids depends on temperature and redox conditions. The CH_4_/C_2_H_6_, CH_4_/C_3_H_8_, and CH_4_/C_4_H_10_ ratios and C concentrations decrease both under isobaric cooling from 1400 to 1200 °С at constant *f*O_2_ and under oxygen fugacity increase from IW-2.5 to IW + 2.5 log units at 1400 °С. As the temperature and water content increase, the concentrations of С_15_-С_18_ alkanes increase slightly while oxygenated hydrocarbons become more diverse. In reduced fluids, only alcohols can reach notable amounts. The fluids with *f*O_2_ IW + 2.5 log units, almost lack methane and contain about 1 rel.% of C_2_H_6_, C_3_H_8_ and C_4_H_10_, as well as C_15–19_ alkanes, and relatively high oxygenated hydrocarbons, especially alcohols and carboxylic acids. The material of capsules causes a catalytic effect: CH_4_/C_2_H_6_ ratios are slightly higher in quenched fluids from Au capsules than in those from Pt capsules. Methanimine (CH_3_N) is the main nitrogen species in the studied fluids, but it loses importance (CH_3_N/N_2_ < 1) at a lower temperature of 1100 °C at constant *f*O_2_ in the system IW-2.5 log units; CH_3_N almost disappears while N_2_ becomes the predominant species as *f*O_2_ decreases to IW + 2.5 log units at 1400 °C.

The behaviour of the CH_3_N species can strongly control the mantle nitrogen cycle, especially in N-poor fluids equilibrated with peridotite. Specifically, silicate phases capable of dissolving ammonia in the presence of NH_3_-bearing fluids hardly can be nitrogen hosts in equilibrium with CH_3_N-bearing fluids. Oxidation of peridotitic fluids with small N contents upon interaction with multiple redox fronts is inferred to decrease strongly the concentrations of methane and methanimine and slightly reduce the amount of ethane, but it causes significant changes neither to C_3_, C_4_ and C_5_ alkanes nor to C_15_-C_19_ alkanes and oxygenated hyrocarbons. As a result, hydrous magma can capture species stable to oxidation, as well as N_2_, which can be involved in diamond formation and carried to shallow mantle.

## Materials and Methods

Starting mixtures for generation of carbon- and nitrogen-bearing fluids consisted of chemical-grade docosane (C_22_H_46_) and stearic acid (С_18_Н_36_O_2_), and 99.9999% pure natural graphite (Table 1). The pre-dried graphite contained 700 ppm CO_2_ and 700 ppm H_2_O determined by chromatography of gases extracted during graphite annealing at 600 °С in a U-shaped quartz cell. Graphite with docosane and/or stearic acid, at a weight ratio of ~10/1, were placed into Pt or Au capsules (Supplementary Fig 1). The 10/1 ratio of graphite to fluid-generating material provided an amount of C-O-H-N fluid sufficient for GC/MS analysis but did not produce overpressure in the capsule after the runs and during drying before gas analysis. Microscopic amounts of nitrogen in the capsules came from air. The samples had different H_2_O concentrations changed by varying the aliquots of stearic acid. Capsules containing starting material for the generation of a C-O-H-N fluid were arc-welded using a *Lampert Werktechnik GmbH* PUK-4U impulse micro welding device. Experiments were carried out in a high-pressure split-sphere multi-anvil apparatus. Fluid was generated at buffered *f*H_2_ in 6.3 GPa runs in a cell with a large low-gradient zone and was analyzed using a Thermo Scientific *DSQ II Series Dual Stage Quadrupole GC/MS* ^34^. Analytical uncertainty for H_2_O, NH_3_, and CO_2_, expressed as precision, was less than 10% and in most cases less than 5% (determined in the range from 12.5 pptv to 12.5 ppbv). The efficiency of the chosen *f*H_2_ buffering technique has been proven in special tests (Supplementary Figs 1–3).

## Electronic supplementary material

Supplementary information

Related Manuscript File
