# Supplementary material for: Carbon and Nitrogen Speciation in N-poor C-O-H-N Fluids at 6.3 GPa and 1100–1400 °C
Source: Sci Rep. 2017 Apr 6;7:706. doi: 10.1038/s41598-017-00679-7 (PMC5428864; doi:10.1038/s41598-017-00679-7)
Supplement: Supplementary file 1 — Supplementary information [file 41598_2017_679_MOESM1_ESM.pdf]

Carbon and Nitrogen Speciation in N-poor C-O-H-N Fluids at 6.3 GPa and 1100-1400°C

Alexander G. Sokol[1,2*], Anatoly A. Tomilenko[1], Taras A. Bul'bak[1], Galina A. Palyanova[1,2], Ivan A. Sokol[1], Yury N. Palyanov[1,2]

[1]*V.S. Sobolev Institute of Geology and Mineralogy, Siberian Branch of the Russian Academy of Sciences, ave. Koptyuga 3, Novosibirsk, 630090 Russia.*

[2]*Novosibirsk State University, str. Pirogova 2, Novosibirsk, 630090 Russia*

*Corresponding author (*sokola@igm.nsc.ru)*

SUPPLEMENTARY INFORMATION

Methods

2.1. High-pressure apparatus

Experiments at 6.3 GPa were carried out in a split-sphere multi-anvil high-pressure apparatus [57]. The multi-anvil sphere of 8/6-type consisted of two anvils with square faces on top and bottom and four side anvils with rectangular faces placed in an octahedral cavity formed by truncating the vertices of eight steel anvils. The sizes of the high-pressure cells were 21.1×21.1×25.4 mm; graphite heaters had inner diameters of 12.2 mm and heights of 18.8 mm. The samples were heated at a rate of 1 deg/s. Pressure was calibrated by recording the change in the resistance of Bi at 2.55 GPa and PbSe at 4.0 and 6.8 GPa at room temperature and by bracketing the graphite-diamond equilibrium at high temperatures. Temperature was monitored in each experiment with a $PtRh_6/PtRh_{30}$ thermocouple calibrated at 6.3 GPa using the melting points of Al, Ag[58]. Pressures and temperatures were measured to the accuracy ± 0.1 GPa and ± 20°C.

2.2 Method of fH_2 buffering

Experimental studies of hydrocarbons at mantle pressures and temperatures face the problem of hydrogen leakage from Pt or Au capsules[30,31,59,60]. Being highly mobile, hydrogen can easily penetrate through the capsule walls made of noble metals and leak rapidly driven by a gradient of hydrogen fugacity (fH_2) between the material of a relatively oxidised high-pressure cell, with the redox conditions controlled by the graphite heater [59], and the H-rich reduced fluid in the capsule. To solve the problem and ensure fH_2 control, we[31] used a modified double-capsule technique: the Pt or Au capsules containing a C-O-H-N fluid were placed into thick-walled Mo capsule with talc and CsCl insulation between the inner and outer capsules (Supplementary Fig. 1a,b). At the experimental pressures and temperatures, talc became decomposed and released water, which reacted with the inner surface of the Mo capsule to produce MoO_2 and hydrogen. The flow of hydrogen between the inner and outer capsules maintained stable fH_2 in the charge. Note that the double-capsule technique with fH_2 set constant by an external H_2 buffer allows changing fO_2 within >3 log units in the inner capsule by varying water concentrations in it (ref. 60). Thus, fO_2 in samples was changed by adding water into the C-O-H-N system.

The modified double-capsule technique was tested in two special runs at 6.3 GPa and 1400°C of very short (1 min, run#1937_2_5) and long (10 hr run#1937_2_5) durations, using a 0.5 mm sample of polycrystalline hematite (Fe_2O_3) as a material sensitive to fH_2 in the inner Pt capsule with graphite (Supplementary Fig. 1c). According to data of unbuffered experiments[61] and theoretical calculations[31], graphite was expected to reduce hematite to the magnetite + wüstite assemblage or to wüstite at these P-T conditions. The phases synthesised in test runs were identified by X-ray powder diffraction on a *Stoe IPDS-2T* diffractometer (MoKα radiation, graphite monochromator) in the Gandolfi mode. Two-

dimensional X-ray patterns were radially integrated using the XArea software package. The diffraction profiles were processed in *WinXPow (Stoe)*. For the phase analysis, the database of PDF-4 Minerals (The Powder Diffraction File PDF-4 +, 2006) was used. The 1-min test run at MMO-buffered fH_2 led to hematite reduction with the formation of predominant magnetite and small amounts of wüstite and hematite (Supplementary Fig. 2). In the 10-hr long run, likewise with fH_2 at MMO, hematite reduced to metallic iron which coexisted with trace amounts of iron carbide and residual hematite (Supplementary Fig. 3). Thus, the test has demonstrated that the outer Mo capsules keep tight during compression/hot-pressing and on during the 10-hr run. This maintains high fH_2 in the outer capsule which provides reduction of the test sample to metallic iron. Note that the same technique was used earlier in runs with durations reaching as long as 40 hours[31].

The furnace assembly used in this study can maintain fO_2 in the Pt capsule with the C-O-H fluid slightly above CW corresponding to low CO_2 content in H_2O-CO_2 fluids[30]. Hydrogen leakage from Pt capsules in unbuffered runs with the Mg_2SiO_4-H_2O-C system leads to the formation of the forsterite-magnesite-graphite/diamond assemblage [62]. Oxygen fugacity in the fluids was estimated from GC-MS data (see below for details of the GC-MS method). Calculations show that buffering by varying the H_2O content in the charges, along with unbuffered runs, allowed fO_2 variations in the range from –2.5 to +2.5 $\Delta\log fO_2$ (IW).

2.3. ***Chromatography–mass spectrometry***

The Pt or Au capsules were placed into a crush cell connected on-line to the gas chromatograph before the analytical column and were heated at 120-130°C for 90 min in a stream of carrier gas (99.9999% pure He). Capsule preparation for analysis included cleaning to avoid contamination. Capsules that did not sustain heating were not analysed. The gas mixture extracted from the capsules

by piercing was analysed by gas chromatography and mass spectrometry using a Thermo Scientific *Focus GS/DSQ II Series Single Quadrupole MS* analyser. The gas mixture was introduced on-line into a stream of He without separation and cryogenic focusing. Gas samples were not pyrolised but heated only to convert water to gas, and the analysis was thus applied to an almost *in situ* gas mixture. Each analytical run was preceded and followed by blank runs.

The gas mixture was separated in a *Restek Rt-Q-BOND* capillary column (100% divinylbenzene as a stationary phase; length 30 m; inner diameter 0.32 mm; film thickness 10 μm). The gas mixture was injected into the analytical column through a 6-port 2-position thermostatic (290°C) *Valco* valve at a constant He flow rate of 1.7 $ml \cdot min^{-1}$, using vacuum compensation; the GC-MS transfer line temperature was 300°C. The temperature program consisted of an isothermal stage (70°C for 2 min) followed by heating ramps of 25°$C \cdot min^{-1}$ to 150°C and 5°$C \cdot min^{-1}$ to 290°C, and the final isothermal stage of 290°C for 100 min. Total ion current (TIC) electron ionisation spectra were collected on a quadrupole mass-selective detector in the full scan mode at 70 eV electron energy and 100 μA emission current. Other conditions were: 200°C ion source; 1350 V multiplier voltage; positive ion detection; 5 to 500 amu mass range; and 563.1 $amu \cdot s^{-1}$ scanning rate. The start time of the analysis was synchronised with crushing.

The collected spectra were interpreted both manually and using *AMDIS 2.72* (Automated Mass Spectral Deconvolution and Identification System) software, with background correction against spectra from the NIST 2014 and Wiley 9 libraries (*NIST MS Search 2.2*, standard search parameters). Peak areas in TIC chromatograms were estimated by the ICIS algorithm Xcalibur (*1.4 SR1 Qual Browser*). The method is suitable for detection of trace volatile concentrations from tens of femtograms. The relative concentrations (rel.%) of volatile components in the studied mixture were obtained by normalisation: the total areas of all chromatographic peaks in the analysed mixture were normalised to 100%, and the areas of individual components defined their shares in the mixture.

The contents of water and principal organic volatiles in gaseous run products were quantified using external standards. For calibration of methane-hexane alkanes, it was injection of certified *Scotty Inc. NL* 34522-PI and 34525-PI gas standards into the gas stream, in the splitless mode, by means of a volumetric gas-tight syringe or a special valve with replaceable loops for volumes from 2 to 500 μL. The calibration quality was checked using the correlation coefficients R^2 in the relationships of peak area vs. injected amount: 0.9975 (16 m/z, n=22) for methane, 0.9963 (26+30 m/z, n=16) for ethane, 0.9986 (29+43 m/z, n=15) for propane, and 0.9994 (29+43 m/z, n=17) for butane. For water, it was either injection of a known amount of deionised distilled water into the gas stream or crushing of the specially prepared Pt and Au capsules with H_2O. In the latter case, the capsules, of the same size as in the experiments, were filled with water, sealed, and welded following the standard procedure. The capsules were weighed before and after crushing, and the weight difference was attributed to released water. The coefficient R^2 for H_2O was 0.9661 (18 m/z, n=7). The concentration ranges of water and alkanes during the calibration were the same as in the run products. Analytical uncertainty was below 5% for C_1-C_4 alkanes (methane to butane) and below 10% for water.

2.4. ***Thermodynamic calculations***

Oxygen fugacity in the C-O-H system at the *P-T* conditions of experiments was calculated by Gibbs free energy minimisation, using the *Selektor-C* software package [63,64] and the thermodynamic database available therein, with g_sprons.db (H_2O, H_2 and O_2), g_Helgeson.db (CH_4, C_2H_6, C_3H_8 and C_4H_{10}) and s_RobieHemingway95 (graphite, diamond). The calculations used a model system with the initial compositions containing the shares of main species (H_2O, CH_4, C_2H_6, C_3H_8 and C_4H_{10}) corresponding to those measured by chromatography–mass spectrometry in the quenched fluids after experiments. The mole fractions of the selected components were estimated with reference to GC-MS calibration results. The starting compositions presumably could contain excess graphite (diamond), as

well as H_2 and O_2, which allowed us to estimate fO_2 in the synthesised fluids at the experimental *P-T* conditions.

Supplementary references

57. Palyanov, Yu.N., Borzdov, Yu.M., Khokhryakov, A.F., Kupriyanov, I.N. & Sokol, A.G. Effect of nitrogen impurity on diamond crystal growth processes. *Cryst. Growth Des.* **10**. 3169-3175 (2010).

58. Sokol, A.G., Borzdov, Yu.M., Palyanov, Yu.N. & Khokhryakov, A. F. High-temperature calibration of a multi-anvil high-pressure apparatus. *High Pressure Research* **35**, 139–147 (2015).

59. Boettcher, A.L., Mysen, B.O. & Allen, J.C. Techniques for the control of water fugacity and oxygen fugacity for experimentation in solid-media high-pressure apparatus. *J. Geophys. Res.* **78**, 5898-5901 (1973).

60. Luth, R.W. Natural versus experimental control of oxidation state: effects on the composition and speciation of C–O–H fluids. *Am. Mineral*. **74**, 50–57 (1989).

61. Frost, D.J., & Wood, B.J. Experimental measurements of the fugacity of CO_2 and graphite/diamond stability from 35 to 77 kbar at 925 to 1650 C. *Geochimica et Cosmochimica Acta*, **61**, 1565-1574 (1997).

62. Sokol, A.G., Kupriyanov, I.N. & Palyanov, Yu.N. Partitioning of H_2O between olivine and carbonate–silicate melts at 6.3 GPa and 1400 °C: implications for kimberlite formation. *Earth Planet. Sci. Lett.* **383**, 58–67 (2013).

63. Karpov, I.K., Zubkov, V.S., Stepanov, A.N. & Bychinskii, V.A. Remake of Chekaluk's thermodynamic model of the system C–H. *Dokl. Akad. Nauk* **358**, 222–225 (1998).

64. Chudnenko, K.V. *Thermodynamic Modelling in Geochemistry: Theory, Algorithms, Software, and Applications* (Academic Publishing House Geo, Novosibirsk, 2010). (in Russ.)

Supplementary figure captions

Fig. S1. **a**. High-pressure cell for studying C-O-H-N fluids at externally buffered fH_2. **b**. Capsules after experimental run. **c**. capsule assembly for testing the modified double-capsule technique. 1 = ZrO_2 container; 2 = cylindrical graphite heater; 3 = $PtRh_6/PtRh_{30}$ thermocouple; 4 = talc; 5 = CsCl; 6 = Pt or Au capsules; 7 = Mo capsule; 8 = MgO; 9 = ZrO_2; 10 = Mo leads; 11 = graphite; 12 = charges with docosane ($C_{22}H_{46}$) and/or stearic acid ($C_{18}H_{36}O_2$); 13 = test sample of Fe_2O_3.

Fig. S2. X-ray powder diffraction pattern of sample 1937_2_5. Vertical lines show the position of peaks (from top to bottom): experimental pattern, Fe_3O_4 (red), Fe_2O_3 (yellow) and $Fe_{0.91}O$ (green).

Fig. S3. X-ray powder diffraction pattern of sample 1939_2_5. Vertical lines show the position of peaks (from top to bottom): experimental pattern, Fe (red), Fe_2O_3 (yellow) and Fe_4C (green).

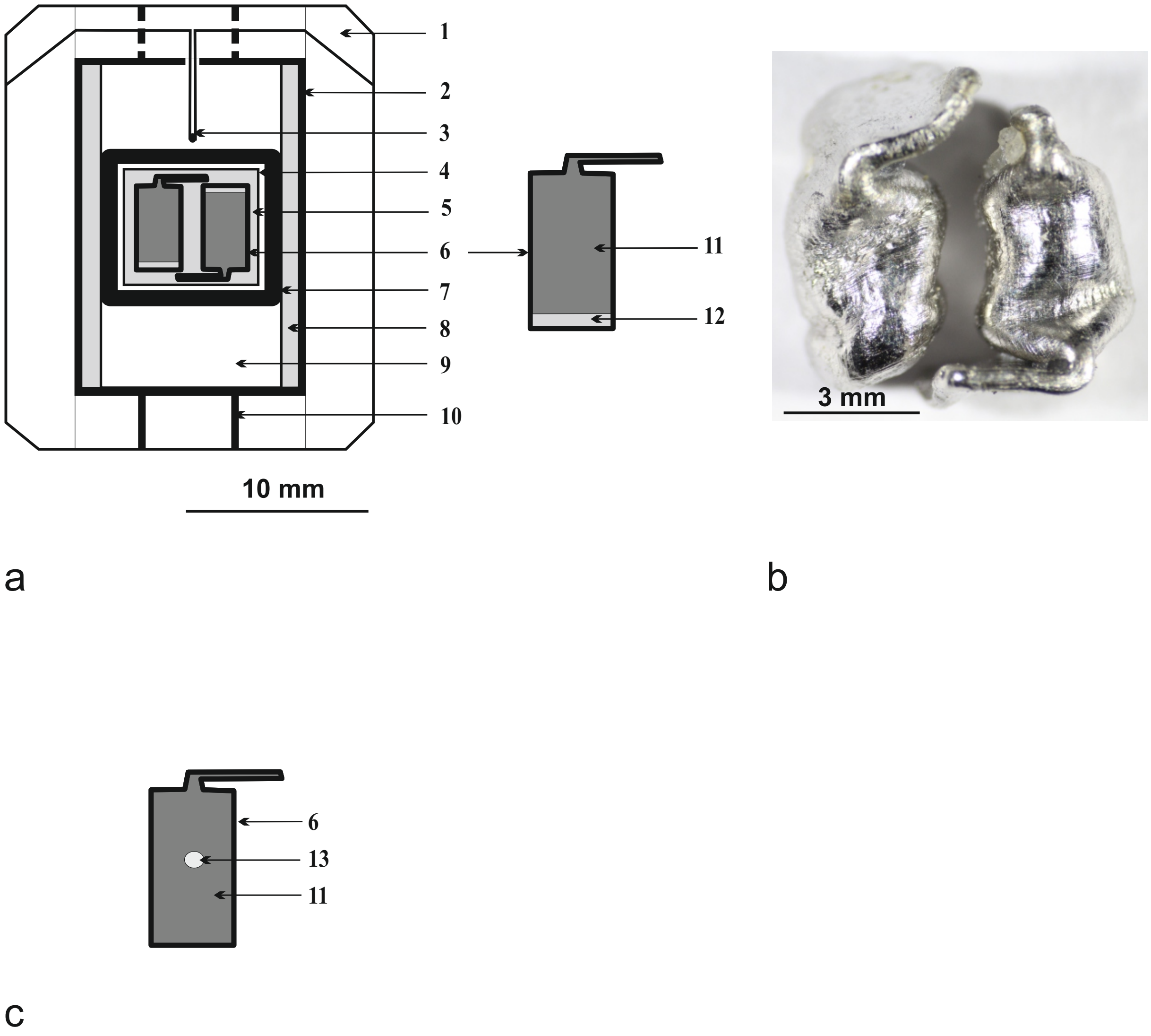

Fig. S1.

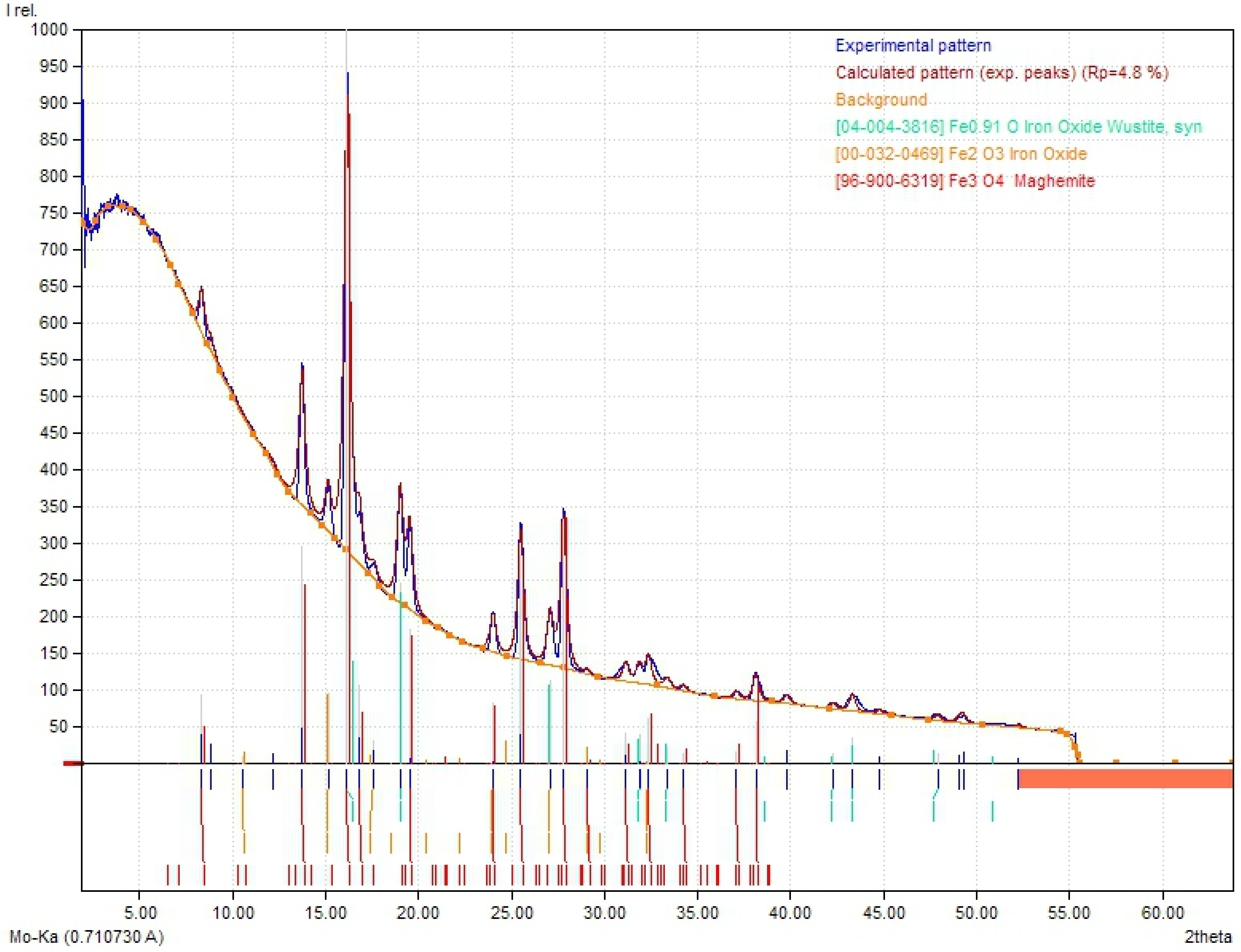
I rel.
1000
950
900
850
800
750
700
650
600
550
500
450
400
350
300
250
200
150
100
50
Experimental pattern
Calculated pattern (exp. peaks) (Rp=4.8 %)
Background
[04-004-3816] Fe0.91 O Iron Oxide Wustite, syn
[00-032-0469] Fe2 O3 Iron Oxide
[96-900-6319] Fe3 O4 Maghemite
5.00
10.00
15.00
20.00
25.00
30.00
35.00
40.00
45.00
50.00
55.00
60.00
Mo-Ka (0.710730 A)
2theta

Fig. S2.

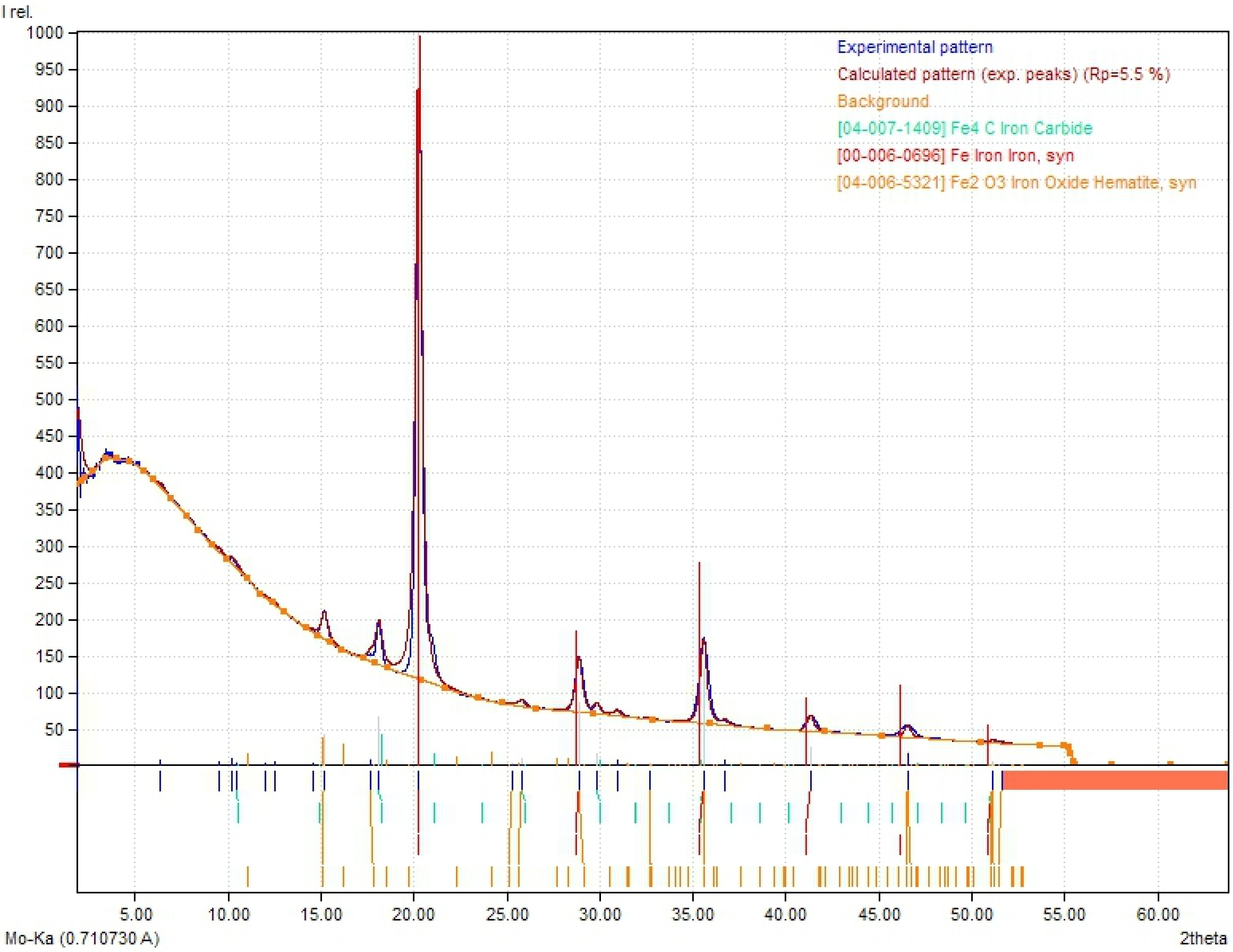
I rel.
Experimental pattern
Calculated pattern (exp. peaks) (Rp=5.5 %)
Background
[04-007-1409] Fe4 C Iron Carbide
[00-006-0696] Fe Iron Iron, syn
[04-006-5321] Fe2 O3 Iron Oxide Hematite, syn
Mo-Ka (0.710730 A)
2theta

Fig. S3.
